# Circadian rhythms in the plant host influence rhythmicity of rhizosphere microbiota

**DOI:** 10.1186/s12915-022-01430-z

**Published:** 2022-10-20

**Authors:** Amy Newman, Emma Picot, Sian Davies, Sally Hilton, Isabelle A. Carré, Gary D. Bending

**Affiliations:** 1 School of Life Sciences, University of Warwick, Gibbet Hill Road, Coventry, CV4 7AL West Midlands UK; 2 Present address: National STEM Learning Centre, University of York, York, YO10 5DD UK; 3 Present address: Micropathology Ltd, Venture Centre, Sir William Lyons Road, Coventry, CV4 7EZ UK

**Keywords:** Bacteria, Circadian rhythms, Fungi, Microbiome, Rhizosphere

## Abstract

**Background:**

Recent studies demonstrated that microbiota inhabiting the plant rhizosphere exhibit diel changes in abundance. To investigate the impact of plant circadian rhythms on bacterial and fungal rhythms in the rhizosphere, we analysed temporal changes in fungal and bacterial communities in the rhizosphere of *Arabidopsis* plants overexpressing or lacking function of the circadian clock gene *LATE ELONGATED HYPOCOTYL* (*LHY)*.

**Results:**

Under diel light–dark cycles, the knock-out mutant *lhy-11* and the gain-of-function mutant *lhy-ox* both exhibited gene expression rhythms with altered timing and amplitude compared to wild-type plants. Distinct sets of bacteria and fungi were found to display rhythmic changes in abundance in the rhizosphere of both of these mutants, suggesting that abnormal patterns of rhythmicity in the plant host caused temporal reprogramming of the rhizosphere microbiome. This was associated with changes in microbial community structure, including changes in the abundance of fungal guilds known to impact on plant health. Under constant environmental conditions, microbial rhythmicity persisted in the rhizosphere of wild-type plants, indicating control by a circadian oscillator. In contrast, loss of rhythmicity in *lhy-ox* plants was associated with disrupted rhythms for the majority of rhizosphere microbiota.

**Conclusions:**

These results show that aberrant function of the plant circadian clock is associated with altered rhythmicity of rhizosphere bacteria and fungi. In the long term, this leads to changes in composition of the rhizosphere microbiome, with potential consequences for plant health. Further research will be required to understand the functional implications of these changes and how they impact on plant health and productivity.

**Supplementary Information:**

The online version contains supplementary material available at 10.1186/s12915-022-01430-z.

## Background

The soil proximal to plant roots, termed the rhizosphere, is a zone of particularly intense microbial activity [1]. Plants exude 3–5% of the carbon (C) that they fix into the rhizosphere, largely as carbohydrates and organic acids [2]. This drives the selective growth of diverse microbial communities, which can have major impacts on plant health. Some rhizosphere microbiota are mutualists and have direct favourable influences upon the plant. For example, mycorrhizal fungi form symbioses with approximately 80% of land plants and improve plants’ uptake of nutrients from the soil [3]. In contrast, microbial pathogens have a direct negative impact on host health, and it is in the interest of the plant to select against colonisation of the rhizosphere by such organisms. Free-living microbes may also interact with the plant indirectly by enhancing nutrient availability, or by inhibiting other microbes such as pathogens [4].

There are considerable qualitative and quantitative differences between root exudates, dependent on plant species, genotype and developmental stage. Exudation is further influenced by a range of biotic and abiotic factors related to both climate and soil properties [5]. As a result, the rhizosphere supports a dynamic microbiome which changes throughout the lifespan of a plant. Remarkably, changes have also been reported to occur over the course of the day-night cycle, including changes in the bacterial composition of the rhizosphere and in the rhizosphere meta-transcriptome [6–9]. Mycorrhizal fungi were also found to exhibit daily rhythms of growth and dieback [10], and key aspects of soil microbial biogeochemical cycling processes such as nitrous oxide emission were shown to display diel rhythmicity [11]. The mechanisms underlying these microbial rhythms are unknown. Many bacteria and fungi possess photoreceptors, and direct responses to light may therefore explain diel rhythmicity of some microbes near the soil surface [12, 13]. Alternatively, these rhythms may be controlled by circadian clocks.

Circadian clocks represent important adaptations to the rhythmic nature of our environment because they allow organisms to adjust their physiology ahead of predictable daily changes in light and temperature conditions linked to the rotation of the earth. In plants, the circadian oscillator drives rhythmic changes in many aspects of metabolism, including photosynthesis, starch biosynthesis and utilisation, nitrogen and sulphur assimilation, and the production of many secondary metabolites [14]. These secondary metabolites include hormones with important roles in shaping plant–microbe interactions, such as abscisic acid, which accumulates in the evening, jasmonic acid, which peaks during the day, and salicylic acid, which peaks during the night [15–18]. Circadian oscillations in the levels of these hormones result in rhythmic changes in immunity to microbial pathogens [19–22]. Circadian rhythmicity is also observed in a range of fungi [23], and the mechanism of the fungal circadian clock has been studied extensively in the model fungus *Neurospora crassa*. While some cyanobacteria are known to exhibit circadian rhythms, and this mechanism is well understood [24], little is known about circadian rhythmicity in non-photosynthetic bacteria such as those found in the plant rhizosphere. Recent evidence suggests that some of these microorganisms may possess endogenous circadian oscillators capable of self-sustained rhythmicity in constant environmental conditions [25–28], while other bacteria may possess hourglass-type clocks, which are driven by rhythmic signals in the environment but incapable of free-running rhythmicity [29].

This raises the question of what happens when microbial timing systems interact with plant circadian rhythms in the rhizosphere. The timing of microbial rhythms relative to those of the plant host may be important to ensure optimal exchange of nutrients between plants and mutualist microbes or to allow pathogens to successfully bypass the plant immune system. Consistent with this hypothesis, altered timing of circadian rhythms in *Arabidopsis* mutants was associated with significantly altered rhizosphere communities [7]. Furthermore, the growth of wild-type plants was impaired following inoculation with the rhizosphere communities of these mutants, suggesting that these changes had negative consequences for plant health [7]. It is therefore important to understand what happens when the circadian clock of the plant host is disrupted.

In order to investigate the impact of plant circadian rhythms on bacterial and fungal rhythms in the rhizosphere, we tested the effect of altered expression of the *LATE ELONGATED HYPOCOTYL* (*LHY)* gene in *Arabidopsis thaliana*. *LHY* functions as part of the negative transcriptional feedback loop that forms the oscillatory mechanism of the plant circadian clock [30]. Under diel light–dark cycles, knock-out mutants (*lhy-11*) exhibit gene expression rhythms that are synchronised to the 24-h cycle, but peak 2–3 h early relative to wild-type plants [31]. On the other hand, *LHY*-overexpressing (*lhy-ox*) plants exhibit driven changes in gene expression in response to dawn and dusk [32].

We first examined the effect of altered plant rhythms under 24-h light–dark cycles where both the *lhy-11* and *lhy-ox* mutants are rhythmic. We then tested whether microbial rhythmicity persisted in *lhy-ox* plants under constant environmental conditions, where these plants become completely arrhythmic. Our results demonstrate that abnormal function of the circadian clock in the plant host alters the range of bacteria and fungi that exhibit rhythmicity in the rhizosphere.

## Methods

### Plant material and growth conditions

The *LHY* knock-out *(lhy-11)* and *LHY*-overexpressing lines (*lhy-1*, referred to here as *lhy-ox*) were in the Landsberg *erecta* (*Ler*) ecotype [33, 34]. Sterile seeds were sown on Murashige and Skoog- agar plates, stratified at 4 °C for 48 h, and grown for 14 days under constant light at 22 °C. Seedlings were then transferred to soil with a 10% moisture content and grown under 12 h of light and 12 h of darkness (12L12D) at 22 °C until harvesting. The soil used in these experiments was a sandy loam Cambisol (pH 6.5, 0.9% organic C) from the Wick series [35]. This was collected from a depth of 0–20 cm from an arable field at Wellesbourne, UK, and sieved using a 5 mm wire sieve.

For dawn vs dusk and time-course analyses under 12L12D, plant roots were harvested together with adhering soil, resulting in a composite sample containing microbes inside roots, on the root surface and in the surrounding rhizosphere. 3 to 4 plants were pooled to generate each sample, and 7 replicates were generated for each time point. Bulk soil (top 1 cm removed) was collected in parallel.

For the time course analysis in constant light, 8 plants were pooled to form one sample, and four such replicate samples were taken at each sampling point. Rhizosphere soil samples were isolated separately from roots. After gentle removal of non-adhering soil, roots were washed twice using 750 µl of sterile water. The two washes were combined to form the rhizosphere compartment for each sample, then immediately frozen using liquid nitrogen. The entire process took under 10 min. Samples were then stored at − 80 °C until nucleic acid extraction.

### Microbial community profiling

DNA and RNA were co-extracted from approximately 0.2 g of root tissue with adhering soil or isolated rhizosphere samples, following a protocol adapted from [36]. The same protocol was applied to bulk soil, starting from 1 g of sample. Samples for rRNA analysis were treated with Amplification Grade DNase I (Sigma-Aldrich, Dorset UK). cDNA was produced using SuperScript III Reverse Transcriptase (Invitrogen, Renfrewshire, UK). Fungal ITS and bacterial 16S rRNA sequences were amplified by PCR from both DNA and cDNA using the ITS primer pair ITS3 and ITS4 [37], and the 16S rRNA primer pair 515f and 806r, respectively [38]. The two primer sets were modified at the 5′ end with Illumina Nextera Index Kit v2 adapters. DNA amplicons were purified using Agencourt AMPure XP beads (Beckman Coulter, Brea, CA, USA) according to the manufacturer’s instructions. The adapted amplicons were then modified by attaching indices and Illumina sequencing adapters using the Nextera XT Index Kit v2 (Illumina, Cambridge, UK) by PCR as described in the manufacturer’s protocol. Amplicons were purified and normalised using the SequalPrep™ Normalization Plate (96) kit (Invitrogen), pooled and sequenced on an Illumina MiSeq using the MiSeq Reagent Kit v3 600-cycle (Illumina).

### Bioinformatics and statistical analyses

Low-quality read ends were removed using Trimmomatic v0.35 [39]. Paired-end reads were assembled by aligning forward and reverse reads using the USEARCH and UPARSE programmes [40, 41]. Primer sequences were trimmed, and sequences filtered to remove low-quality reads (-fastq_maxee 0.5). Unique sequences were then sorted by abundance, with singletons discarded, and assigned to Operational Taxonomic Units (OTUs) at a 97% minimum identity threshold. These were then filtered for chimeras using the GOLD database for 16S rRNA and the UNITE database for ITS [38, 42]. Quantitative Insights into Microbial Ecology (QIIME) 1.9.1 [43] was used to assign taxonomy using the Greengenes [44] and UNITE databases for 16S rRNA and ITS, respectively. 23.1 million reads were retained from the 16S rRNA samples and 15.1 million reads for ITS samples, for use in subsequent analyses.

Samples with below 1000 reads were discarded. Remaining samples were normalised using DESeq2 [45], then numbers of reads were converted to relative abundance. Ordination plots and analysis of similarity (ANOSIM) were performed using the vegan package [46] in R. Fungal OTUs were annotated using information from the FUNGuild database [47], in order to assign trophic modes and functional guilds. Manual filtering was carried out to ensure that only well-known examples would be retained for analysis. Kruskal–Wallis tests with Dunn post hoc tests were used to determine significant differences between reads assigned to fungal guilds.

### Analysis of microbial rhythmicity

ANOSIM was used to test for significant differences between the overall composition of fungal and bacterial communities in dawn vs dusk comparisons, and Kruskal–Wallis pairwise testing was used to compare the relative abundance of different OTUs. For time-course experiments, data were filtered to eliminate very low abundance OTUs that were detected in less than 50% of time points. Rhythmic changes in abundance of individual OTUs were then detected using the MetaCycle package, which integrates three well-established methods, ARSER (ARS), JTK_CYCLE (JTK) and Lomb-Scargle (LS) to provide a single *p* value for rhythmicity [48]. For the light–dark dataset, OTUs were identified as rhythmic when a period value was identified between 18 and 30 h, with a Benjamini-Hochberg-corrected *p* value (BHQ) less than 0.01. The constant light dataset was more highly replicated and less noisy, so a higher BHQ threshold was applied (0.05). MetaCycle analysis of permuted versions of the data failed to identify rhythmic patterns. This showed that rhythms detected within our data were robust and not caused by stochastic variation.

### Analyses of plant gene expression

RNA was extracted from root samples using the [36] method. Expression of the *CCA1* and *LUX* transcripts was analysed by quantitative PCR using an Agilent Mx3005P detection system and SYBR Green JumpStart Taq Readymix (Sigma-Aldrich) according to the instructions of the manufacturer. Expression levels were calculated relative to the constitutively expressed gene *ACT2* (At3g18780) using the 2^−deltaCT^ method.

## Results

### The plant clock gene LHY influences the recruitment of fungal and bacterial microbiota into the root and rhizosphere

We first tested whether aberrant function of the *LHY* gene affected the microbial composition of the rhizosphere. Wild-type, *lhy-11* and *lhy-ox* plants were grown in agricultural sandy loam soil under diel light–dark cycles composed of 12 h of light and 12 h of darkness (12L12D). Samples consisting of roots and adhering rhizosphere were harvested at dawn and dusk on a single day. The composition of the microbiome was assessed through both ribosomal RNA (rRNA) and ribosomal DNA (rDNA) profiling. rRNA was used to characterise the transcriptionally ‘active’ fraction of the microbiome, whereas rDNA was used to characterise the ‘total’ communities that accumulate in the root and rhizosphere over the lifetime of plants, including dead, dormant and live members (Emerson et al., 2017). Sequences were assigned to groups of closely related organisms commonly described as operational taxonomic units (OTUs), which are used as proxies for microbial species. Within each rhizosphere sample, the abundance of sequences matching each OTU was quantified relative to total sequence numbers, to give an estimate of the relative abundance or activity of each OTU as a proportion of total bacterial or fungal communities.

**Fig. 1:**
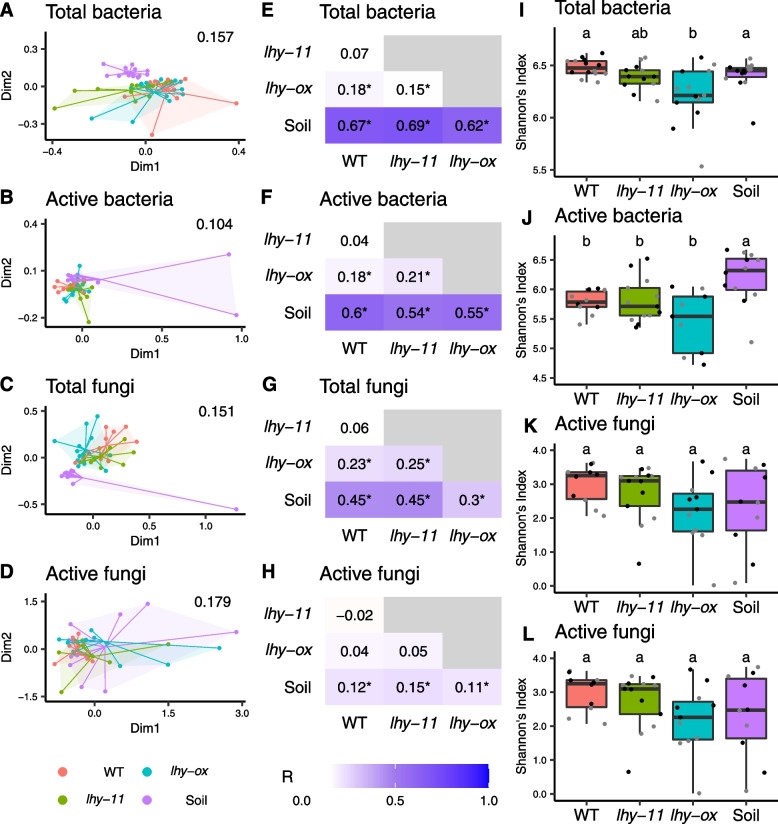
Impact of the *lhy-ox* and *lhy-11* mutations on the rhizosphere microbiome. Taxonomic analyses of ITS and 16S rRNA sequences were carried out from DNA and RNA samples, in order to determine the composition of total and metabolically active bacterial and fungal communities. **A**–**D** show NMDS plots with stress values indicated. Each data point represents an individual sample. Lines connect data points to the centroid for each group of samples. Stress values are indicated in the top-right corner. **E**–**H** show analyses of similarity (ANOSIM) plots. Darker colours indicate increasing dissimilarity. Numbers are dissimilarity scores (R) and * indicates *p* < 0.05. **I–L** show analyses of alpha diversity, quantified using Shannon’s index. Different letters indicate significantly different diversity scores, as determined by Kruskal–Wallis followed by pairwise Wilcoxon rank sum tests. Grey points indicate morning samples; black evening samples

We first investigated differences in overall microbial community composition between the different plant genotypes. Morning and evening samples were combined for these analyses. This revealed significant differences in both bacterial and fungal communities, between *lhy-ox* and wild-type plants and between *lhy-ox* and *lhy-11* plants (Fig. 1 A–H). Similar results were obtained based on analyses of active or total microbial communities, with the exception of active fungi, where no significant differences were detected between plant genotypes (Fig. 1D, H).

Analyses of total microbial communities showed that the diversity of bacterial and fungal species present (alpha diversity, quantified using Shannon’s index) was significantly reduced in the *lhy-ox* rhizosphere relative to wild-type and *lhy-11* plants (Fig. 1I, K). While species richness did not significantly differ, species evenness was significantly reduced in *lhy-ox* samples relative to wild-type and *lhy-11* plants (Additional File 2: Table S1). These results suggest that the level of *LHY* gene expression in the plant host influences the relative abundance of different microorganisms rather than the range of species present.

**Fig. 2:**
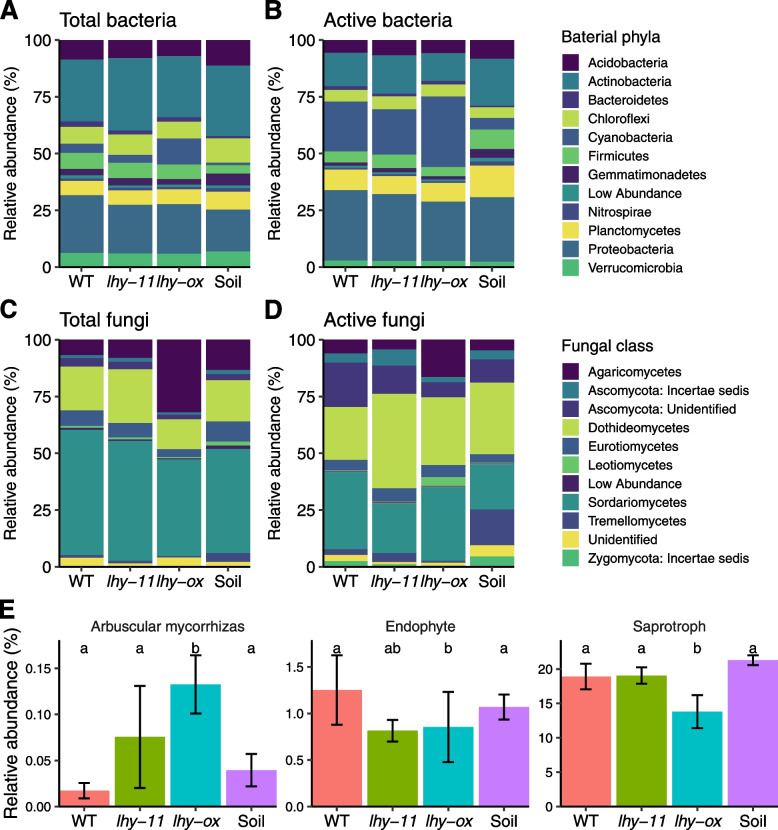
Altered composition of the rhizosphere microbiome in *lhy-ox* and *lhy-11* mutants. **A**–**D** Relative abundance of bacterial phyla and fungal classes in the rhizosphere, based on analyses of total or active communities. **E** Relative abundance of fungi annotated as arbuscular mycorrhizas, endophytes, and saprotrophs. Ecological guild information for the OTUs was obtained from the FUNGuild database. Data are means and standard errors from 14 samples

Consistent with this observation, several bacterial and fungal phyla differed in relative abundance between wild-type, *lhy-11* and *lhy-ox* samples (Fig. 2A, C). Actinobacteria and Chloroflexi were significantly more abundant in *lhy-11* samples than in wild-type samples (*p* = 0.003 and 0.022, respectively), while Bacteroidetes and Proteobacteria were less abundant (*p* = 0.046 and 0.08, respectively). The relative abundance of Acidobacteria and Proteobacteria was reduced in the *lhy-ox* rhizosphere relative to the wild-type rhizosphere (*p* = 0.046 and 0.027, respectively), and that of Chloroflexi, Gemmatimonadetes and Actinobacteria was reduced relative to *lhy-11* samples (*p* = 0.026, 0.013 and 0.006, respectively). Compared with wild-type or *lhy-11* samples, *lhy-ox* samples were also found to contain significantly higher levels of Agaricomycetes (*p*<0.001 for both comparisons), and lower levels of Dothideomycetes (*p* = 0.020 and *p* < 0.001, respectively) and Eurotiomycetes (p = 0.022 and p = 0.012, respectively). Sordariomycetes were less abundant in *lhy-ox* than *lhy-11* samples (p = 0.013). Fewer significant differences were identified based on the analysis of active microbial communities (Fig. 2B, D), but differences in the relative abundance of Proteobacteria (wild-type vs *lhy-ox*) and Agaricomycetes (*lhy-11 *vs *lhy-ox*) were confirmed (*p* = 0.045 and *p* = 0.027, respectively). In addition, a large reduction in Tremellomycetes was observed in *lhy-ox* samples relative to wild-type and *lhy-11* samples (Fig. 2B, D; *p* = 0.027 and *p* = 0.041).

These results demonstrated that overexpression and loss-of-function of the *LHY* gene alter the relative abundance of different bacterial and fungal taxa in the root and rhizosphere.

### The *lhy-ox* and *lhy-11* mutations alter the relative abundance of fungal guilds with relevance for plant health

To investigate whether the altered composition of the rhizosphere microbiome in *lhy-ox* and *lhy-11* mutants may have consequences for plant health, fungal OTUs were assigned to ecological guilds based on the FUNGuild database [47]. Analyses of total fungal communities revealed that *lhy-ox* samples contained a higher relative abundance of arbuscular mycorrhizal fungi than wild-type and *lhy-11* samples (*p* = 0.0017 and *p* = 0.0065, respectively). On the other hand, *lhy-ox* samples contained a lower relative abundance of endophytes than wild-type samples (*p* = 0.029), and a lower relative abundance of saprotrophs than both wild-type and *lhy-11* samples (*p* = 0.034 and *p* = 0.047, respectively) (Fig. 2E).

While the overall abundance of fungal pathogens was not significantly affected, specific pathogenic OTUs showed significant differences in abundance between plant genotypes. Analyses of total fungal communities revealed that *LHY* overexpression was associated with a 5- to 7-fold reduction in abundance of *Plectosphaerella *sp. in the rhizosphere relative to both the wild-type and *lhy-11*, and about twofold reduction in abundance of *Dendryphion nanum*. *Cladosporium exasperatum* and *Alternaria *sp. were also reduced about twofold in *lhy-ox* relative to *lhy-11*, and *Fusarium neocosmosporiellum* about sixfold relative to the wild-type (Table 1, A). Largely similar trends were observed based on analyses of active rhizosphere communities, with overexpression of *LHY* being associated with a lower relative abundance of these pathogens (Table 1, B).

**Table 1:** Effect of knock-out or over-expression of *LHY* in the plant host on the relative abundance of pathogenic OTUs in the rhizosphere

	**Wild-type rhizosphere**	***lhy-11* rhizosphere**	***lhy-ox* rhizosphere**	**Soil**
**(A) Mean relative abundance in total communities (%)**				
*Cladosporium exasperatum*	5.0 (ab)	*7.9* (b)	3.7 (a)	7.2 (ab)
*Plectosphaerella *sp.	8.5 (a)	6.6 (a)	1.3 (b)	2.1 (a)
* Alternaria *sp.	2.4 (ab)	3.0 (b)	1.4 (a)	3.5 (b)
*Dendryphion nanum*	2.2 (a)	1.6 (a)	0.9 (b)	0.5 (b)
*Fusarium neocosmosporiellum*	1.8 (a)	0.4 (ab)	0.3 (b)	0.7 (a)
**(B) Mean relative abundance in active communities (%)**				
*Plectosphaerella *sp.	6.4 (a)	2.9 (ab)	2.6 (b)	0.3 (ab)
*Alternaria *sp.	5.3 (a)	5.1 (a)	1.6 (b)	4.7 (ab)
*Dendryphion nanum*	5.2 (a)	6.1 (a)	3.9 (ab)	2.4 (b)
*Devriesia *sp.	2.0 (a)	0.7 (b)	1.0 (ab)	0.7 (b)
*Fusarium neocosmosporiellum*	1.2 (a)	0.7 (a)	0.1 (b)	1.2 (ab)

Different letters indicate significant differences (*p* < 0.05)

These results showed that both overexpression and loss of function of *LHY* led to changes in abundance of both beneficial and pathogenic fungi in the plant rhizosphere.

### Loss of function or overexpression of LHY results in altered rhythmicity of rhizosphere microbes under diel light–dark cycles

Morning and evening samples were then compared to each other to assess diel changes in rhizosphere composition. This revealed no significant differences in overall microbial community structure, but differences in relative abundance of individual OTUs were identified using Kruskal–Wallis pairwise testing. Full lists of OTUs showing dawn vs dusk differences are provided as Additional File 1. Largely distinct sets of OTUs were identified as showing dawn vs dusk differences based on analysis of active or total communities (Additional File 2: Fig. S1), suggesting that these different analyses may provide complementary information about bacterial and fungal rhythmicity in the rhizosphere.

Analysis of total communities in the rhizosphere of wild-type *Arabidopsis* plants revealed dawn vs dusk differences in about 5% of bacterial OTUs (Table 2, A). This was lower but comparable to the previous estimate of 13% [49]. Whereas this previous study reported a threefold reduction in rhythmicity of bacterial OTUs in the rhizosphere of plants with disrupted clock function (*CCA1-ox*), we found that the *lhy-ox* and *lhy-11* rhizospheres contained a similar proportion of rhythmic OTUs to wild-type plants when maintained under diel light–dark cycles (Table 2, A). However, analysis of active bacterial communities identified significantly more dawn vs dusk differences in *lhy-11* samples than in WT and *lhy-ox* samples. Consistent with previous observations [49], rhythmic bacterial OTUs belonged to a range of phyla including Proteobacteria, Acidobacteria, Actinobacteria, and Firmicutes, but also Chloroflexi, Cyanobacteria, Gemmatimonadetes, Planctomycetes, and Verrucomicrobia (Additional File 2: Fig. S2 A-H).

**Table 2:** Frequency of bacterial and fungal OTU rhythmicity under diel light–dark cycles

	**Bacteria**		**Fungi**	
	**Total number**	**% rhythmic**	**Total number**	**% rhythmic**
**(A) Total OTUs**				
**Wild-type**	4328	4.7 (a)	373	4.0 (a)
***lhy-11***	4226	4.5 (a)	416	2.6 (a)
***lhy-ox***	4260	4.6 (a)	427	7.7 (b)
**Soil**	4067	4.0 (a)	487	3.3 (a)
**(B) Active OTUs**				
**Wild-type**	3854	2.9 (a)	172	3.5 (a)
***lhy-11***	3943	5.3 (b)	170	5.3 (a)
***lhy-ox***	3742	2.3 (a)	154	5.8 (a)
**Soil**	3883	1.6 (c)	169	0.0 (b)

Total (A) and active (B) rhizosphere communities were analysed through sequencing of 16S and ITS rDNA and rRNA, respectively. Rhythmic changes in relative abundance of individual OTUs were detected based on comparison of dawn and dusk samples. See also Additional File 1 for lists of rhythmic OTUs detected in this experiment. Different letters indicate significant differences (*p* < 0.05)

Within fungi, significantly more OTUs were found to show dawn vs dusk differences in *lhy-ox* samples than in wild-type or *lhy-11* samples, based on analyses of total but not active communities. Most rhythmic fungal classes belonged to the phylum Ascomycota, including Sordariomycetes, Dothideomycetes, Leotiomycetes and Eurotiomycetes. Others belonged to the Basidiomycota (Agaricomycetes) (Additional File 2: Fig. S2 I-O).

**Fig. 3:**
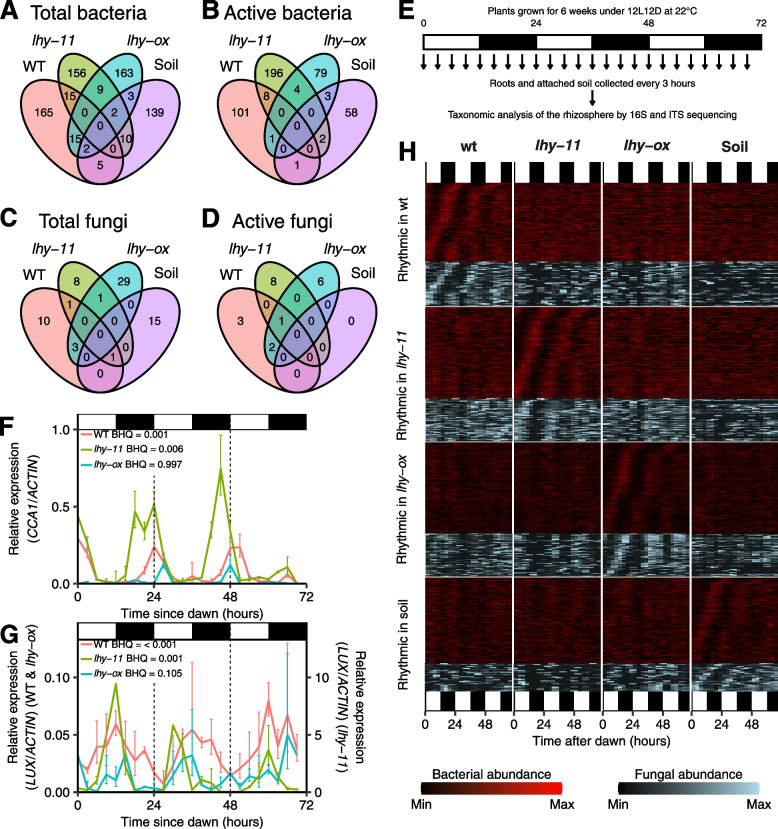
Rhythmicity of bacterial and fungal OTUs under diurnal light–dark cycles. **A**–**D** Venn diagrams comparing OTUs identified as rhythmic based on comparison of dawn and dusk samples in the rhizosphere of different plant genotypes or in soil, based on analyses of total or active microbial communities. Lists of rhythmic OTUs detected in this experiment are provided as Additional File 1. **E** Experimental design for time-course sampling. **F**, **G** Expression of the circadian clock genes *CCA1* and *LUX* in root samples from the time course. White and shaded areas indicate days and nights, respectively. BHQ values indicate Benjamini–Hochberg corrected p values for rhythmicity. **H** Raster plots displaying temporal changes in the relative abundance of individual bacterial and fungal OTUs in soil and rhizosphere samples. Each rectangle represents the relative abundance of one OTU at a particular time point, from one sample derived from 3 to 4 plants. A moving 2-point average was applied to reduce noise in the data. Each row of panels shows OTUs that were detected as rhythmic by MetaCycle in the rhizosphere of wild-type, *lhy-11* and *lhy-ox* plants or in bulk soil, based on period values between 18 and 30 h, and BHQ < 0.01. Each column shows temporal patterns for these same OTUs in the other samples. OTUs were sorted according to period length and phase in the sample in which they were detected as rhythmic. White and black rectangles below the chart indicate days and nights respectively. Lists of rhythmic OTUs detected in this experiment are provided as Additional File 3

The microbiota that showed dawn vs dusk differences in the rhizosphere of the different plant genotypes were largely distinct, and no bacterial or fungal OTU was scored as rhythmic in the rhizosphere of all 3 plants, i.e., wild-type, *lhy-ox* and *lhy-11* (Fig. 3A–D). There was also little overlap between OTUs that were detected as rhythmic in bulk soil and in rhizosphere samples. This did not reflect the presence or absence of these OTUs, because approximately 90% of rhythmic OTUs were detected in all sample types (Additional File 2: Fig. S3). There were also differences in the taxonomic distribution of rhythmic OTUs, with for example Chloroflexi, Agaricomycetes and Leotiomycetes showing more frequent rhythmicity in wild-type samples, and Firmicutes and Dothideomycetes in *lhy-ox* samples, based on analyses of total communities (Additional File 2: Fig. S2).

One possible explanation for different microbiota being identified as rhythmic in the rhizosphere of the two mutants was that aberrant circadian clock function in the *lhy-11* or *lhy-ox* mutants resulted in altered timing of bacterial and fungal rhythms. This would have resulted in rhythmic patterns that were no longer detectable based on comparison of dawn and dusk time points, while other rhythms would be revealed. A time-course experiment was therefore carried out to test this hypothesis (Fig. 3E–H). Plants were grown under 12L12D cycles for 7 weeks, then combined root and rhizosphere samples were harvested every 3 h over 72 h (Fig. 3E). Single samples were harvested at each time point, each derived from 3 to 4 plants. Circadian clock function in the plant roots was tested by assaying expression of the plant clock genes *CCA1* and *LUX* (Fig. 3F, G). Both genes showed the expected rhythmic pattern of expression in wild-type root samples, peaking at dawn and at dusk, respectively. On the other hand, the *lhy-11* and *lhy-ox* mutants both showed abnormal timing and amplitude of these rhythms. Active fungal and bacterial communities were then profiled based on ITS and 16S rRNA sequences at every time point. A moving 2-point average was applied to reduce noise in the data, and the rhythm analysis software MetaCycle [48], which integrates the outcomes of 3 independent rhythm detection algorithms into a single *p* value, was used to identify rhythmic changes. OTUs were identified as rhythmic when a period value was identified between 18 and 30 h, with a Benjamini-Hochberg-corrected *p* value (BHQ) less than 0.01 (Additional File 3). We are confident that this stringent analysis did not detect random fluctuations in the data because only one OTU was detected as rhythmic when the dataset was randomised, but it remains possible that a few OTUs were wrongly scored as rhythmic due to the high level of noise in the data. We have provided the full list of OTUs down to BHQ = 0.05, together with the timecourse data, so that others can make up their own assessment.

In wild-type samples, different rhythmic OTUs peaked at different times of the day-night cycles, forming an ecological succession (Fig. 3H). However, the rhythmic patterns observed for these OTUs were lost in the rhizosphere of the *lhy-11* and *lhy-ox* mutants, as well as in soil. On the other hand, other OTUs exhibited rhythmic patterns in these samples, but were arrhythmic in the wild-type rhizosphere. There was also very little overlap between bacterial and fungal OTUs that were rhythmic in soil or in the plant rhizosphere, indicating that the plant root promotes the rhythmic behaviour of a different set of microbes (Fig. 3A–D; H).

This confirmed the observations from the comparison of dawn and dusk time points and showed that the altered function of the *LHY* gene in the plant host did not simply alter the timing of bacterial and fungal rhythms in the rhizosphere. Instead, it amplified rhythmicity in some microbes while suppressing that of others, resulting in temporal reprogramming of the rhizosphere.

### Rhythmicity under constant light in the rhizosphere of wild-type plants

**Fig. 4:**
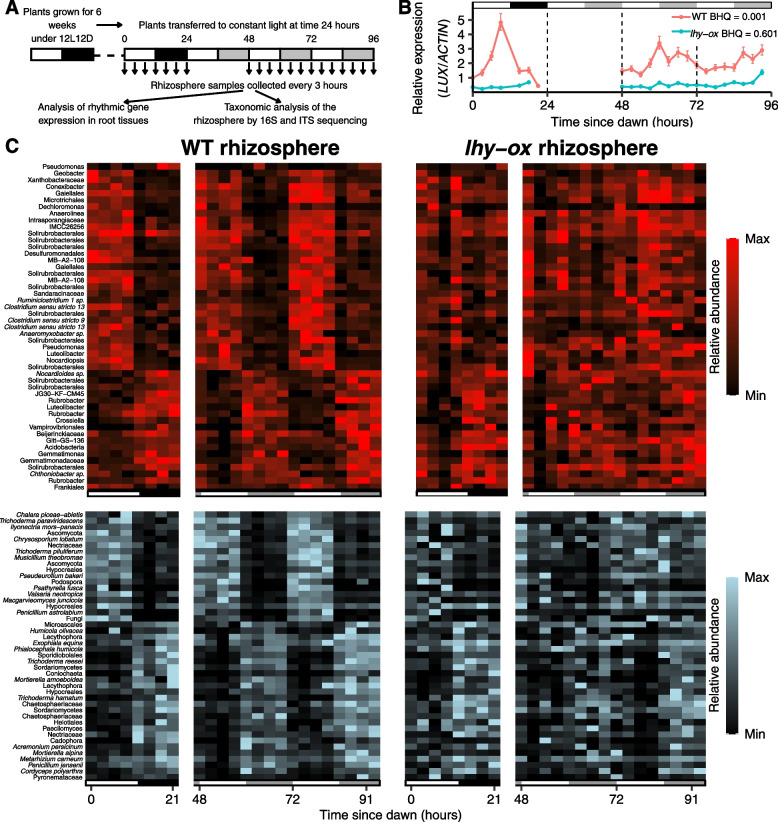
Rhythmicity of bacterial and fungal OTUs in constant conditions. **A** Experimental design. Plants were grown for 6 weeks under 12L12D cycles before sampling started. Four replicate root and rhizosphere samples were collected every 3 h, each derived from 8 plants. After 24 h, plants were transferred to constant light. Sampling was resumed at time 48 h for another 2 days, in order to test for free-running rhythmicity in constant conditions. **B** Temporal expression patterns of the *LUX* mRNA in wild-type and *lhy-ox* mutant roots. Benjamini–Hochberg p-values (BHQ) are given. **C** Temporal changes in relative abundance of individual bacterial OTUs (red) and fungal OTUs (blue) in constant light. Rhythmic OTUs were identified based on period values between 18 and 30 h and BHQ < 0.05. Left-hand panels show their rhythmic changes in the rhizosphere of wild-type plants. Right-hand panels show disrupted rhythmicity for the same OTUs in the rhizosphere of *lhy-ox* plants. Each rectangle represents the relative abundance of one OTU at a particular time point, averaged from 4 replicate samples each composed of 8 plants. White and black rectangles below the chart indicate days and nights, respectively. Grey rectangles indicate subjective nights in constant light conditions. Lists of rhythmic OTUs detected in this experiment are provided as Additional File 4

A fundamental property of circadian rhythms is that they persist, or free-run, when the organism is transferred to constant environmental conditions. In order to test whether bacterial and fungal rhythms in the rhizosphere were driven by daily light–dark cycles or whether they reflected the activity of a circadian clock, plants were grown under diel light–dark cycles composed of 12 h of light and 12 h of darkness (12L12D) for 6 weeks, then transferred to constant light. Rhizosphere samples were collected every 3 h during the last day-night cycle, then over 48 h in constant light (Fig. 4A). 4 replicate samples were collected per time point, each composed of 8 plants. Total fungal and bacterial communities were profiled based on ITS and 16S rDNA sequences at every time point. OTUs that exhibited rhythmic changes in abundance over the constant light portion of the experiment were then identified using MetaCycle [48].

Wild-type root samples showed the expected, rhythmic pattern of *LUX* gene expression, peaking in the evening, and this rhythm persisted upon transfer to constant light conditions (Fig. 4B). About 1.6% of bacterial OTUs and 12% of fungal OTUs exhibited rhythmicity in the absence of environmental time cues (Table 3). While bacterial OTUs that were rhythmic in constant light represented a relatively small proportion of the overall rhizosphere community in terms of abundance (6.2%), rhythmic fungal OTUs represented over 42%, suggesting extensive rhythms of fungal growth and death at the plant-soil interface.

**Table 3:** Frequency of bacterial and fungal OTU rhythmicity in constant light

Total OTUs	Bacteria		Fungi	
	**Total number**	**% rhythmic**	**Total number**	**% rhythmic**
**Wild-type**	2972	1.6	376	12
***lhy-ox***	2691	0.1	281	1.1

Total rhizosphere communities were analysed through sequencing of 16S and ITS rDNA. Rhythmic changes over the 48-h timecourse were detected using MetaCycle, based on period values between 18 and 30 h, and Benjamini–Hochberg corrected *p* values BHQ less than 0.05. See also Additional File 4 for lists of rhythmic OTUs detected in this experiment

Fungal and bacterial rhythms in constant light were highly similar in phase to those observed under the light–dark portion of the experiment (Fig. 4C). Temporal patterns fell into two clear clusters, with OTU abundance peaking either during the day or during the night. This contrasted with the broad spectrum of rhythms detected in the previous experiment under light–dark cycle (Fig. 3H); however, this may either result from the different type of sample (isolated rhizosphere in this experiment, as compared to combined root and rhizosphere) or from the different type of analysis (total communities in this experiment as compared to active communities). Rhythmicity was identified within multiple fungal phyla, including Ascomycetes, Basidiomycetes and Mortierellomycetes. It was also found within a broad range of bacterial families, including Acidobacteria, Actinobacteria, Chloroflexi, Cyanobacteria, Firmicutes, Gemmatimonadetes, Proteobacteria and Verrucomicrobia (Additional File 2: Table S2).

This persistence of OTU rhythmicity under constant environmental conditions demonstrated control by a circadian clock. However, it remained unclear whether this clock belonged to the plant host, or whether individual microbes possessed intrinsic clocks capable of self-sustained rhythmicity in the absence of environmental time cues.

### Disrupted rhythmicity under constant light in the rhizosphere of arrhythmic *lhy-ox* plants

In order to test whether rhythmic changes in the abundance of rhizosphere microbiota under constant light required rhythmicity of the plant host, we tested whether bacterial and fungal rhythms persisted under constant conditions in the rhizosphere of *lhy-ox* plants, which become arrhythmic in the absence of environmental time clues [33]. Expression of the *LUX* gene was arrhythmic in *lhy-ox* roots in constant light, consistent with disrupted function of the plant circadian clock (Fig. 4B). This was associated with disrupted rhythmicity for most of the fungal and bacterial OTUs that were rhythmic in the wild-type rhizosphere (Fig. 4C, D). In contrast to what was observed under light–dark cycles, only two bacterial OTUs and no fungal OTUs appeared to gain rhythmicity in *lhy-ox* samples in constant light, and approximately 10 times fewer bacterial and fungal OTUs were identified as rhythmic in constant light in the *lhy-ox* rhizosphere compared to the wild-type rhizosphere (Additional File 4, Table 3). This demonstrated that function of the plant circadian clock is required for rhythmicity of most bacterial and fungal OTUs in the rhizosphere.

**Fig. 5:**
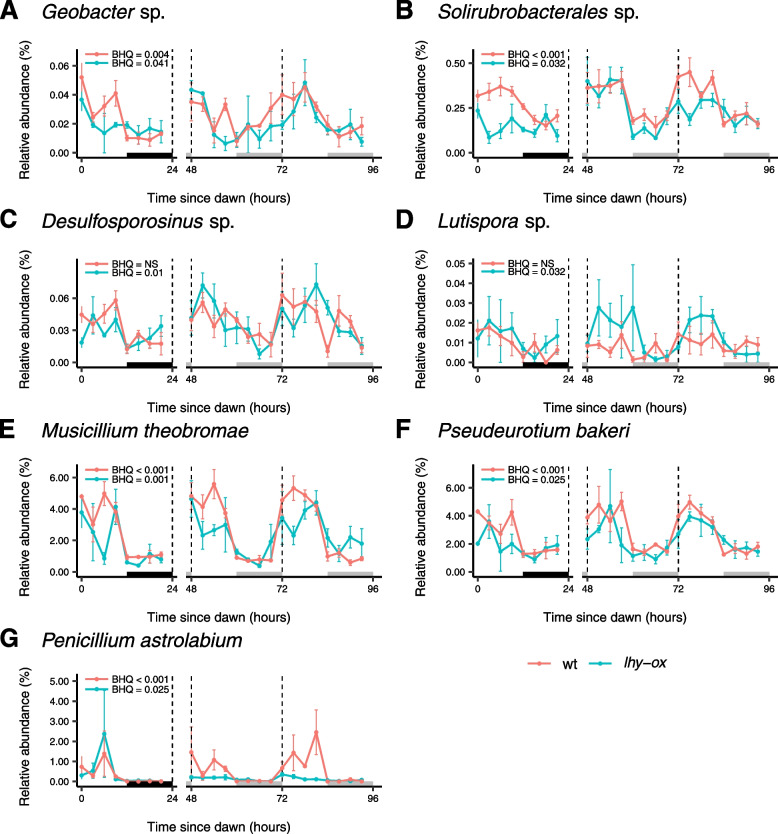
Rhythmic bacterial and fungal OTUs in the *lhy-ox* rhizosphere. Individual graphs show rhythmic changes in relative abundances of individual OTUs. Each data point corresponds to the mean of 4 samples, each derived from at least 8 plants. Error bars indicate standard errors. Benjamini–Hochberg corrected *p* values (BHQ) are shown

On the other hand, 4 bacterial OTUs and 3 fungal OTUs exhibited free-running rhythmicity in the rhizosphere of *lhy-ox* plants, suggesting the presence of endogenous timing mechanisms in these microbes (Fig. 5). Within bacteria, this included a member of *Solirubrobacterales,* and species of *Geobacter, Lutispora* and *Desulfosporosinus*. Rhythmic fungal OTUs were identified as the probable plant pathogen *Musicillium theobromae*, the probable saprotroph *Pseudeurotium bakeri*, and the possible saprotroph *Penicillium astrolabium*. *Lutispora* sp. and *Desulfosporosinus* sp. were significantly rhythmic in *lhy-ox* samples but not in the wild-type rhizosphere, suggesting that their autonomous rhythmicity may be suppressed in the rhizosphere of plants with functional circadian clocks. Alternatively, the arrhythmic plants may produce signals that invoke rhythmicity, rather than relieving suppression of rhythmicity. On the other hand, *Penicillium astrolabium* displayed higher amplitude rhythms and higher relative abundances in wild-type samples, suggesting that root-derived signals may act to boost its circadian rhythmicity (Fig. 5G).

## Discussion

### Altered microbial rhythmicity in the rhizosphere of circadian clock mutants

Our results add to the growing evidence that some fungi and bacteria possess true circadian oscillators, which are capable of free-running rhythmicity in the absence of environmental time cues [25, 26]. However, they also demonstrate that the rhythmicity of rhizosphere microbiota can be influenced by the circadian clock of the plant host.

Under 12L12D cycles, the extent of fungal and bacterial rhythmicity was comparable between the *lhy-ox* and the wild-type rhizospheres, reflecting the rhythmicity of both plant genotypes under these conditions. However, the loss of rhythmicity of *lhy-ox* plants upon transfer to constant environmental conditions was associated with loss of rhythmicity of about 90% of rhizosphere microbiota. Our observations are consistent with the hypothesis that a functional plant circadian clock is required for the rhythmicity of rhizosphere microbiota. This suggests that circadian rhythms in plant roots may drive bacterial and fungal rhythms in the rhizosphere, through rhythmic exudation of metabolites and/or defence molecules. Changes in humidity levels in the soil surrounding the roots may also drive cycles of microbial growth and death, as circadian changes in plant water uptake have been reported [50–52].

Other explanations are possible for the effect of the *lhy-ox* mutation, however. Mis-regulation of the clock in plants is known to lead to alterations in root development [53, 54]. *lhy-ox* plants exhibited fewer lateral roots than wild-type and *lhy-11* plants in our experiment, although this was not quantified. Different clock mutants are also known to exhibit different water use efficiencies [55], which may lead to different levels of moisture around the roots. Either of these factors could also account for disruption of microbial rhythmicity in the *lhy-ox* mutant rhizosphere, although there is no simple explanation for why this effect would be more prominent under constant conditions than under diel light–dark cycles.

Further experiments would be required to demonstrate coupling between microbial and plant rhythms, such as testing whether the period of fungal and bacterial rhythms is altered in the rhizosphere of a period mutant. Such experiments may prove to be difficult, however, because altered function of the circadian clock in the short-period mutant *lhy-11* led to rhythmicity of distinct microbiota under diel light–dark cycles. The *lhy-11* mutation resulted in a 3-h advance of *CCA1* and *LUX* gene expression and was anticipated to cause a similar advance of microbial rhythms in the rhizosphere. Instead, this was associated with rhythmicity of a completely different set of bacterial and fungal OTUs. Distinct microbiota were also found to exhibit rhythmicity in the rhizosphere of *lhy-ox* mutants under diel light–dark cycles, suggesting once again the complete temporal reprogramming of the rhizosphere.

One potential explanation for this result is that the *lhy-11* and *lhy-ox* mutations affect not only the timing of transcriptional rhythms downstream of the clock, but also their amplitude. Loss of function and overexpression of *LHY* would be expected to have opposite effects on the level of expression of LHY-target genes, and to amplify the oscillation of some pathways downstream of the clock while suppressing that of others. This may in turn alter both the overall composition and the temporal changes in root exudates, which may suppress rhythmicity of some microbiota while eliciting that of others. While previous work only uncovered the rhythmic secretion of 7 compounds from *Arabidopsis* roots [56], a more recent study detected rhythmicity of 50 exudates [57]. A largely distinct complement of root exudates showed diel rhythmicity in the rhizosphere of circadian clock mutants *toc1-101* and *cca1-1* mutants [57]. We therefore hypothesise that rhythmicity of different exudates in the *lhy-11* and *lhy-ox* rhizospheres may explain the rhythmicity of different sets of microbiota.

### Functional consequences for the plant rhizosphere

The *lhy-ox* mutation was associated with significantly different bacterial and fungal community structures relative to wild-type and *lhy-11* mutant plants. While altered root morphology and/or altered water efficiency in the *lhy-ox* mutant may have resulted in the selection of different bacterial and fungal populations compared to wild-type and *lhy-11* plants [53–55], our findings are consistent with previous observations that altered function of the plant circadian clock can result in selection of a distinct microbial community [7, 58].

While the short-period mutation *toc1-21* was previously associated with a significant reduction in species richness and evenness in the rhizosphere relative to the Ws wild-type [58], we found that another short-period mutation, *lhy-11*, had no significant impact on the microbial community structure. Altogether, these observations suggest that the manner in which circadian clock mutations impact on the root microbiome cannot simply be predicted from the period phenotype of the mutant.

Interestingly, the *lhy-ox* mutation was found to impact on the relative abundance of several fungal guilds. For example, fungi classified as endophytes were present at a reduced level in the rhizosphere of *lhy-ox* plants. These symbiotic fungi live within plant tissues and can benefit plants in exchange for carbohydrates, by increasing water or nutrient uptake, or by producing secondary metabolites that act as deterrents for herbivores [59]. Because of their intimate interaction with plant tissues, they are directly exposed to rhythmic changes driven by the plant circadian clock, and appropriate coordination with metabolic rhythms of the plant host may be important for optimal growth of these microorganisms.

On the other hand, arbuscular mycorrhizal (AM) fungi benefit many plants by forming symbiotic interactions with plant roots, but they cause growth impairment in *Arabidopsis* plants [60]. These fungi were more abundant in the roots of *lhy-ox* plants, suggesting that elevated expression of *LHY* favours recruitment of these detrimental microorganisms to the *Arabidopsis* rhizosphere. Furthermore, the recruitment of these fungi into *lhy-ox* plants may in fact have been underestimated in our analyses, because primer choice can influence the precise microbial community amplified, and Glomeromycota, which form arbuscular mycorrhizas, are thought to be under-represented when ITS primers are used for microbial community profiling rather than 18S rRNA primers [61]. Both loss of function and overexpression of *LHY* were also associated with changes in the relative abundance of different pathogens. While these effects of *LHY* loss of function or overexpression may be independent of its role in circadian timing, they are consistent with the hypothesis that plant clock function affects recruitment of fungal microbiota to the rhizosphere, with potential impacts for plant health and productivity.

## Conclusions

Our results demonstrate that circadian rhythms in the plant root orchestrate the rhythmicity of microbial communities in the rhizosphere. This is potentially important for agriculture and horticulture, because domestication and cultivation of crops at a broad range of latitudes has in many cases led to selection of specific alleles of circadian clock genes [62]. Further research will be required to fully understand whether, and how, rhythmicity of different microbes in the rhizosphere of circadian clock mutants impacts on plant health and performance in the field. Changes in microbial rhythmicity may for example alter the temporal patterns of biogeochemical processes and affect availability of key nutrients at times of the day when plants are able to utilise them. Future work should therefore aim to uncover how microbial gene function varies over the course of the day, how this is coordinated with the plant transcriptome, and how this coordination is altered in the rhizosphere of plants with altered rhythmicity.

## Supplementary Information

**Additional file 1.** Lists of bacterial and fungal OTUs identified as rhythmic in dawn vs dusk comparisons under 12L12D.

**Additional file 2:** **Table S1.** Comparison of fungal and bacterial species richness in the rhizosphere of wild-type, lhy-ox and lhy-11 plants. **Table S2.** Taxonomy of rhythmic bacteria and fungi in the plant rhizosphere under constant light conditions. **Fig. S1.** Analyses of total and active microbial communities identify distinct sets of rhythmic OTUs. **Fig S2.** Presence of fungal and bacterial OTUs across samples. Fig. S3. Rhythmic changes in the relative abundance of bacterial phyla and fungal classes under light-dark cycles.

**Additional file 3.** Lists of bacterial and fungal OTUs identified as rhythmic through analysis of a 72-hour time-course under 12L12D.

**Additional file 4.** Lists of bacterial and fungal OTUs identified as rhythmic through analysis of a 48-hour time-course in constant light.

## Data Availability

Raw sequence files were uploaded to the NCBI (National Centre for Biotechnology Information) Sequence Read Archive (https://www.ncbi.nlm.nih.gov/sra) under the BioProject accession numbers PRJNA804715 for the dawn vs dusk comparison [63], PRJNA804001 for the time-course experiment under light–dark cycles [64] and PRJNA678539 for the time-course experiment in constant light [65].
