# Supplementary material for: Circadian rhythms in the plant host influence rhythmicity of rhizosphere microbiota
Source: BMC Biol. 2022 Oct 20;20:235. doi: 10.1186/s12915-022-01430-z (PMC9585842; doi:10.1186/s12915-022-01430-z)
Supplement: Supplementary file 2 — Additional file 2: Table S1. Comparison of fungal and bacterial species richness in the rhizosphere of wild-type, lhy-ox and lhy-11 plants. Table S2. Taxonomy of rhythmic bacteria and fungi in the plant rhizosphere under constant light conditions. Fig. S1. Analyses of total and active microbial communities identify distinct sets of rhythmic OTUs. Fig S2. Presence of fungal and bacterial OTUs across samples. Fig. S3. Rhythmic changes in the relative abundance of bacterial phyla and fungal classes under light-dark cycles. [file 12915_2022_1430_MOESM2_ESM.docx]

**SUPPLEMENTARY INFORMATION FOR:**

**Circadian rhythms in the plant host influence rhythmicity of rhizosphere microbiota**

Amy Newman, Emma Picot, Sian Davies, Sally Hilton, Isabelle A. Carré and Gary D. Bending

**Table S1: Comparison of fungal and bacterial species richness in the rhizosphere of wild-type, *lhy-ox* and *lhy-11* plants.** Unplanted soil is shown as a control. Values shown are the mean number of OTUs per sample ± standard error. Different letters indicate significant differences (p < 0.05 as determined by Kruskal-Wallis followed by pairwise Wilcoxon rank sum tests).

|  | **Total communities** | | **Active communities** | |
| --- | --- | --- | --- | --- |
|  | **Number of bacterial OTUs** | **Number of fungal OTUs** | **Number of bacterial OTUs** | **Number of fungal OTUs** |
| **WT** | 2234 ± 103 a | 160 ± 12 a | 1821 ± 98 a | 70 ± 7 a |
| ***lhy-11*** | 2056 ± 90 a | 177 ± 11 a | 1816 ± 73 a | 57 ± 10 a |
| ***lhy-ox*** | 2136 ± 95 a | 191 ± 11 a | 1797 ± 85 a | 46 ± 10 a |
| **Soil** | 2025 ± 51 a | 312 ± 24 b | 1915 ± 173 a | 52 ± 14 a |

**Table S2: Taxonomy of rhythmic bacteria and fungi in the plant rhizosphere under constant light conditions.** The table lists taxonomic groups in which a fraction of OTU members were found to exhibit rhythmicity in the rhizosphere of wild-type or *lhy-ox* plants in constant light, based on the experiment in Figure 4.

| 1. **Bacterial phyla** | Wild type rhizosphere | | *lhy-ox* rhizosphere | |
| --- | --- | --- | --- | --- |
|  | Number of OTUs | % rhythmic | Number of OTUs | % rhythmic |
| Acidobacteria | 226 | 0.4 | 226 | 0.0 |
| Actinobacteria | 522 | 4.5 | 478 | 0.2 |
| Chloroflexi | 233 | 1.3 | 209 | 0.0 |
| Cyanobacteria | 15 | 6.7 | 18 | 0.0 |
| Firmicutes | 165 | 2.4 | 136 | 1.5 |
| Gemmatimonadetes | 64 | 3.1 | 59 | 0.0 |
| Proteobacteria | 803 | 1.1 | 736 | 0.1 |
| Verrocomicrobia | 142 | 2.1 | 129 | 0.0 |

| 1. **Fungal phyla** | Class | WT rhizosphere | | *lhy-ox* rhizosphere | |
| --- | --- | --- | --- | --- | --- |
|  |  | Number of OTUs | % rhythmic | Number of OTUs | % rhythmic |
| Ascomycota | Dothideomycetes | 39 | 2.6 | 32 | 0.0 |
|  | Eurotiomycetes | 28 | 14.3 | 16 | 6.2 |
|  | Leotiomycetes | 22 | 22.7 | 18 | 5.5 |
|  | Pezizomycetes | 6 | 16.7 | 3 | 0.0 |
|  | Sordariomycetes | 145 | 17.9 | 113 | 0.9 |
| Basidiomycota | Agaricomycetes | 22 | 4.5 | 11 | 0.0 |
|  | Microbotryomycetes | 10 | 0.1 | 8 | 0.0 |
| Mortierellomycota | Mortierellomycetes | 14 | 14.3 | 15 | 0.0 |

**Figure S1: Analyses of total and active microbial communities identify distinct sets of rhythmic OTUs.** Venn diagrams comparing OTUs identified as rhythmic in total communities (pink) or in active communities (blue).

**Figure S2: Rhythmic changes in relative abundance of bacterial phyla and fungal classes under light-dark cycles.** Rhythmic taxa were identified based on comparison of dawn and dusk samples. Total and active microbial communities were analysed based on sequencing of rDNA and rRNA respectively. Data represent mean relative abundance values from 7 replicate samples, each comprising 3-4 plants.


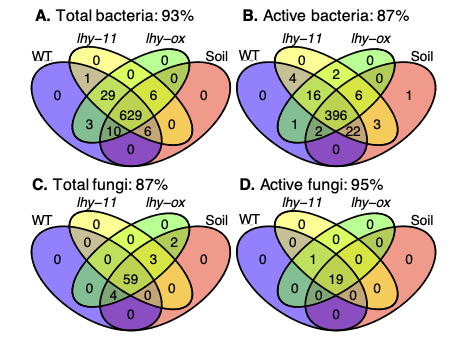
**Figure S3: Presence of fungal and bacterial OTUs across samples.** These Venn diagrams compare the presence of OTUs that were detected in the rhizophere of any plant genotype or in soil, across all sample types. This shows that the majority of these OTUs were common to all samples.
